# Photosensitivity Reactions Induced by Photochemical Degradation of Drugs

**DOI:** 10.34172/apb.2022.010

**Published:** 2021-05-29

**Authors:** Hajnal Klelemen, Gabriel Hancu, Edina Kacsó, Lajos-Attila Papp

**Affiliations:** Department of Pharmaceutical Chemistry, Faculty of Pharmacy, University of Medicine, Pharmacy, Science and Technology “George Emil Palade” of Târgu Mureș, Târgu Mureș, Romania.

**Keywords:** Photochemical degradation, Photochemistry, Phototoxicity, Photoallergy, UV radiation

## Abstract

Photochemical degradation of drugs can lead to degradation products with potential toxic or allergizing effects for the human body. A significant amount of work has been carried out over the past few decades to clarify the molecular mechanism of photosensitizing processes observed after the administration of certain drugs and exposure to light. There is a close relation between the photosensitizer effect of a drug and its chemical structure. Compounds possessing certain moieties and functional groups in their molecular structure, like aromatic chromophore systems or photo-dissociable bonds that can form free radicals, and consequently are susceptible to have light-induced adverse effects. Photoionization, photodissociation, photoaddition and photoisomerization are the main chemical processes, which can occur during the photochemical decomposition of a pharmaceutical compound. The current study is a short review describing photochemical degradation of certain pharmaceuticals, presenting specific examples from various pharmaceutical classes for the different types of decomposition mechanisms. In vivo methods and clinical tests available for the investigation of photosensitizing reactions are also discussed.

## Introduction

The terms *phototoxicity* and *photoallergy* refer to pathophysiological conditions that result from the combined action of light and a chemical agent.^1-3^

The organ primarily affected by photochemical drug damage is the skin. Neither light nor drug alone can induce photochemical reactions; however, a combination of the two factors in certain “favourable” conditions can. The type and severity of symptoms that occur are determined by the photo reactivity of the drug molecule. Photosensitivity symptoms may occur in every skin type.^4-6^

*Phototoxicity* is a common phenomenon, it occurs at higher doses of the drug, and is usually rapid (within 24 hours, but sometimes symptoms can appear in a few minutes); it occurs at first application and is limited to areas exposed to sunlight. The symptoms usually resemble to sunburns. The inflammatory reaction results from direct cellular damage induced by a photochemical reaction between the chemical photosensitizing agent and the radiation. In the case of *photoallergies*, the effect is not dose-dependent, it can occur at low doses, but only after repeated administration. This is usually a delayed hypersensitivity reaction that occurs in 24-72 hours after drug exposure.^5-7^

Under the influence of ultraviolet (UV) radiation, the drug, or its photochemical degradation product acts as an allergen, attaching to proteins on the epidermal cells, generating antigens that sensitize the surrounding lymphocytes. Individuals previously sensitized in this way may experience symptoms after repeated exposure to light, not only in the irradiated area. Photoallergic reactions usually occur in the form of itchy eczema rashes and are similar in appearance to allergic contact dermatitis.^8-12^

If it is determined that these reactions are clearly caused by the drug, treatment should be discontinued as more severe symptoms may develop. However, in some cases the symptoms considered to be caused by phototoxicity and photoallergy may be triggered by other factors, so establishing the exact cause for of the symptomatology is actually a difficult task.^8^

It is important to mention *photophobia* as a separate problem. In the case of photophobia, the patient avoids the light because the eyes are sensitive to it and the light effect causes pain. Carcinogenicity has also been reported in several cases of photosensitizing agent’s administration.^7,13^

The active substances which demonstrated the potential to induce photosensitivity reaction are listed in different official databases. Data are summarized based on results from *in vitro* (photostability and photosensitivity tests) and *in vivo* assays. The most important guidelines are summarized in the S10 ICH Guideline “Photosafety Evaluation of Pharmaceuticals” and the FDA S10 “Photosafety Evaluation of Pharmaceuticals, Guidance for Industry”.^14,15^

The most frequently mentioned active ingredients in the literature responsible for photosensibility are summarized in the Table 1.^16-20^

**Table 1:** Drugs generating photosensitivity^16-20^

**Therapeutic group**	**Drugs**
Central nervous system (CNS) acting agents	*Neuroleptics*: chlorpromazine, thioridazine, trifluoperazine, fluphenazine, flupentixol, thiothixene, chlorprothixene, haloperidol, clozapine *Antidepressants*: amitriptyline, imipramine, fluoxetine, paroxetine, sertraline, venlafaxine *Sedato-hipnotics*: diazepam, alprazolam, chlordiazepoxide *Antiepileptics*: carbamazepine, lamotrigine, phenytoin
Cardiovascular system acting agents	*Antiarrhythmics*: amiodarone, quinidine *Ca*^2+^* channel blockers*: nifedipine, amlodipine, diltiazem *ACE inhibitors*: ramipril, quinapril, enalapril *Diuretics*: furosemide, hydrochlorothiazide *Statins:* simvastatin, atorvastatin, pravastatin
Non-steroidal anti-inflammatory drugs	Ketoprofen, ibuprofen, naproxen, diclofenac, indomethacin, phenylbutazone
Antidiabetics	Glipizide, glyburide, glibenclamide, glimepiride
Antihistamines	Cyproheptadine, diphenhydramine, dimetindene, loratadine, cetirizine
Antipathogenic agents	*Antibacterial agents:* fluoroquinolones, tetracyclines, sulphonamides and trimethoprim, cefotaxime, ceftazidime, metronidazole *Antifungals*: voriconazole, itraconazole, terconazole, flucytosine, griseofulvin, bifonazole, fluconazole *Anti-malaria agents*: quinine, chloroquine, hydroxychloroquine *Antivirals:* efavirenz, saquinavir, acyclovir, ritonavir, zalcitabine
Antineoplastic agents	Vandetanib, imatinib, fluorouracil and structurally related substances (tegafur, capecitabin), paclitaxel, methotrexate, vinblastine, dacarbazine
Systemic dermatologic agents	Isotretinoin, methoxsalen, acitretin
Herbs	Hypericin, fluorocoumarin

Because of their widespread use, phenothiazine neuroleptics, amiodarone, furosemide and hydrochlorothiazide, aril propionic acid non-steroidal anti-inflammatory drugs, fluoroquinolones or tetracyclines can be mentioned among others to exhibit significant photosensitizing effect.^21-25^

Our review, compiles lists of drugs reported to cause photochemical reactions, and systematically review the evidence for this association.

## Physico-chemical background of photochemical reactions

According to the first law of photochemistry (Grotthuss-Draper), only the light that is absorbed by a given molecule can trigger a photochemical reaction. According to the second law of photochemistry (Stark-Einstein), a quantum absorbed photon can cause a physical and/or a chemical change in a molecule. Under the Lambert-Beer law, the extent of light absorption is proportional with the concentration of the substance.^26-28^

From the statements mentioned above, the following logical conclusions can be drawn:

- Direct contact with light is essential for photochemical reactions to occur.
- An arbitrary light source cannot produce any change; photosensitivity of drugs depends on UV-VIS light absorption pattern of drugs.
- High luminance and prolonged irradiation result in the absorption of more photons, so the degree of photochemical decomposition is proportional to the intensity of the light effect.
- At a given luminance, the magnitude of the reaction is proportional to the concentration.
- The first step in the interaction between molecules (M) and light (h*ν*) is the absorption of the photon, which means excitation of the molecule and uptake of excess energy:

M + h*ν* = M *

- Although excitation of the molecule is a prerequisite for reactions, this does not always happens because the molecule can emit the energy absorbed as electromagnetic radiation. As far the former does not occur, then the molecule is chemically degraded.
- What happens is a function of the strength of the chemical bonds in the molecule. Based on the above, the following situations are possible^29,30^:
- *Light emission*: M* → M + h*v*, the molecule is reset from the excited state while emitting light, in which case the possibility of a chemical reaction is eliminated.
- *Intermolecular Energy Transfer*: M* + N → M + N*, where N* is the excited molecule, while the M molecule remains chemically unchanged. Typically, these situations occur between aromatic polycycles and nucleotide bases, which increase their propensity for photoaddition reactions, and may react with a compound involved in energy transfer.
- *Photoionization*: M* → M⁺˙ + ē, which usually means radical formation and occurs when the molecule was not originally of a radical structure. This radical can participate in further reactions, oxidize other molecules, being very reactive.
- *Photodissociation*: M* → N˙ + P˙, which represents a homolytic splitting of bonds, resulting in the formation of two radicals, which are also very reactive during photoionization.
- *Photoaddition*: M* + N → M-N, which occur between the drug molecule and a biomolecule, however, photo adducts of the drug molecule with non-biomolecules may be toxic.
- *Photoisomerization*: M* → N, during which intramolecular rearrangement of covalent bonds occurs; it can also result in toxic products.^19^

## Role of functional groups of drug molecules in photo reactivity

**Figure 1:**
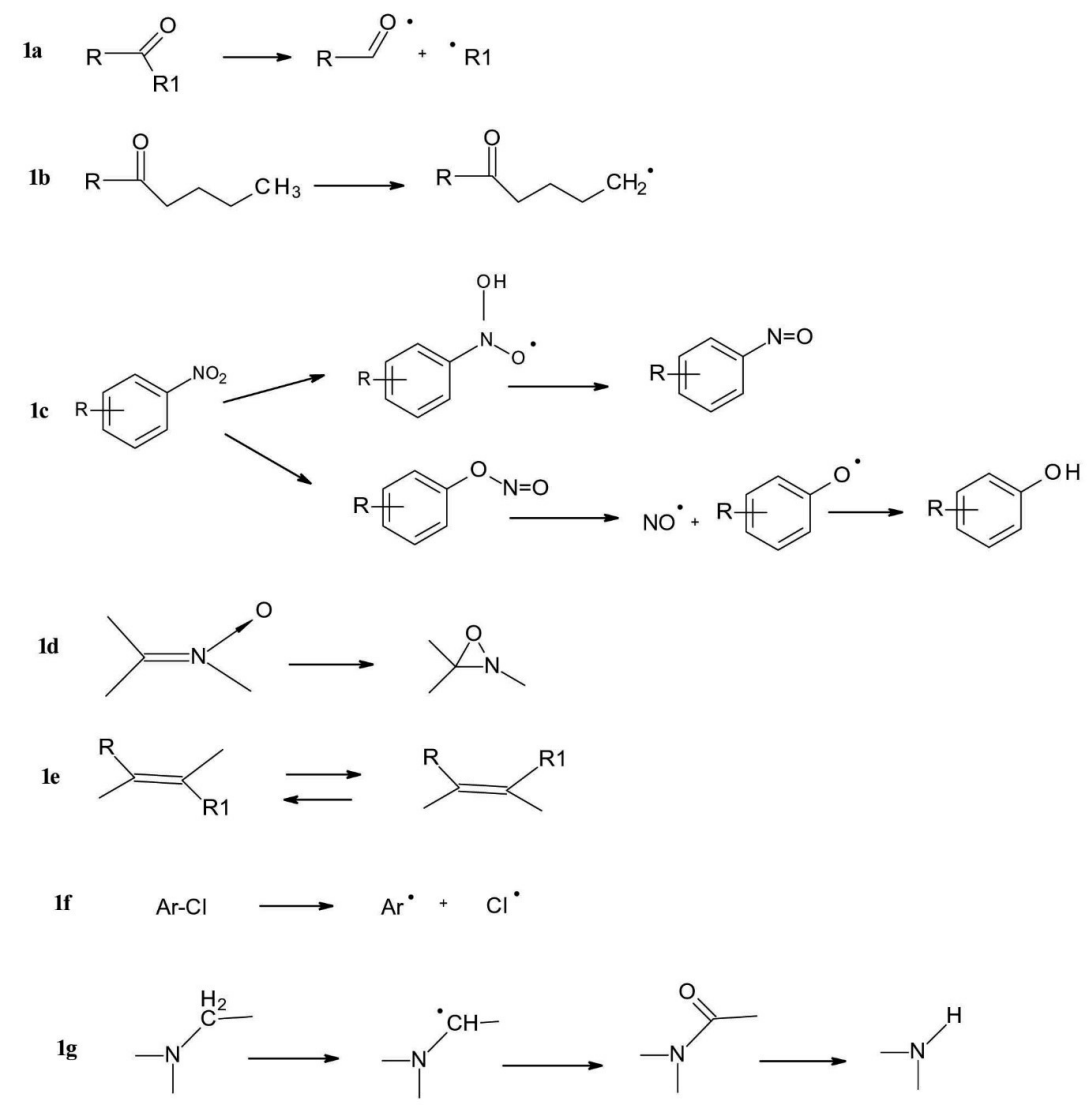
Typical photochemical transformations of some common chemical groups.

A high degree of photochemical reactivity is displayed by drugs that are readily excited by light; in general, this assumes a π-electron system and / or an oxidable heteroatom (S) or easy to split single bonds.^31^

a. *Carbonyl group* behaves as an electrophilic radical in the π* excited state. Typical reactions are reduction via intermolecular hydrogen abstraction and fragmentation either via radical cleavage of the C-C bond between a carbonyl carbon atom and a carbon atom in a α position (“Norrish Type I” reaction) (Figure 1a) or via intramolecular decomposition of the γ-position in a C-H bond which reacts with the carbonyl group, resulting in a hydroxyl group formation and the formation of two carbon atom radicals (“Norrish Type II” reaction) (Figure 1b).

b. *Nitroaromatic group*, also behave as a radical, undergoing intermolecular hydrogen abstraction or rearrangement to a nitrite ester. Reactions of the aromatic nitro group include the reduction of the nitro group to the nitroso group, wherein the reducing agent is a hydrogen from a solvent or another molecule. Another reaction which can occur is the rearrangement of nitric acid to an ester, which is subsequently decomposed with formation of nitric oxide and phenoxy radicals, followed by formation of phenols (Figure 1c).

c. *N-oxide function* rearranges easily to an oxaziridine compound and the final products often result from further reaction of this intermediate (Figure 1d).

d. *C=C double bond*, liable to *E/Z*isomerization as well as to oxidation (Figure 1e).

e. *Aryl chloride* is liable to homolytic and/or to heterolytic dichlorination. Other photochemical reactions of aromatic and heteroaromatic systems include dehalogenation when the aromatic ring-substituted components undergo transformation or reduction during irradiation (Figure 1f).

Dehalogenation reactions also include the replacement of the halogen atom in the aqueous medium with a hydroxyl group. During the substitution of the halogen atom, photo hydrolysis reactions can be observed, the most important groups of drugs which undergo such conversion are phenothiazine derivatives (e.g., chlorpromazine), and thiazide diuretics (e.g., chlorothiazide, hydrochlorothiazide).

f. Products containing a *weak C-H bond,* e.g. in a *benzylic position* or in α position related to an *amine*group. These compounds often undergo photoinduced fragmentations via hydrogen transfer or electron-proton transfer (Figure 1g).

**Figure 2:**
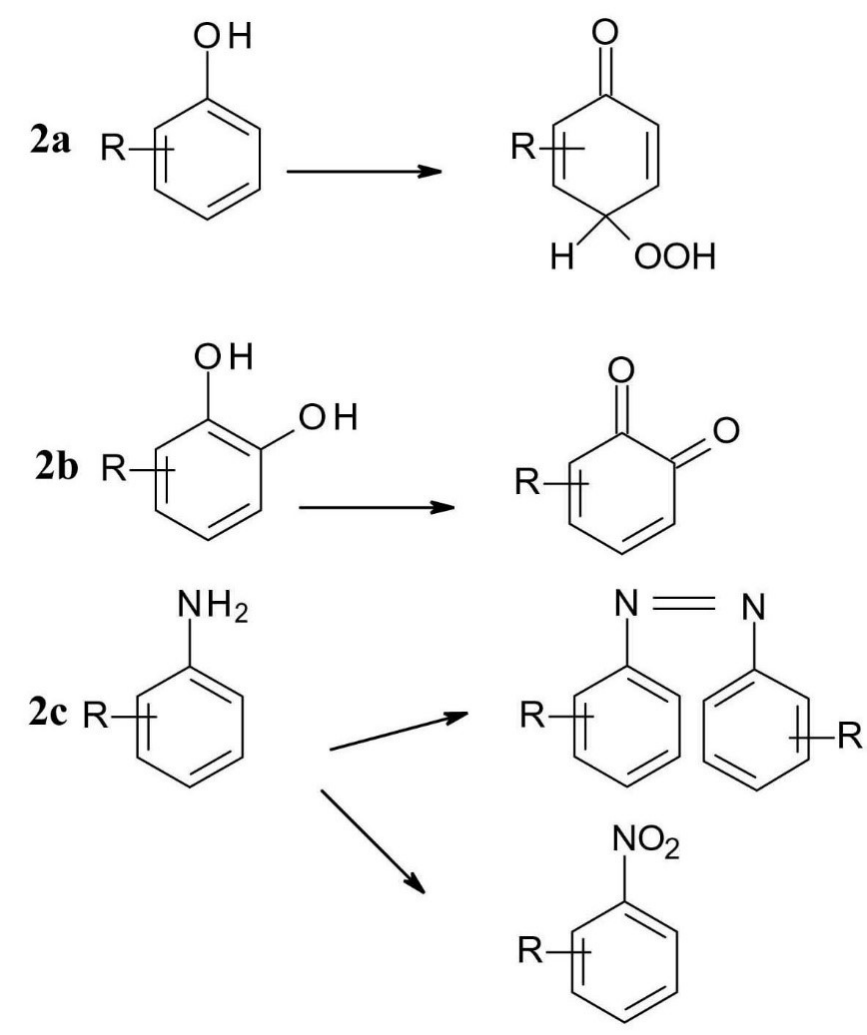
Photochemical degradation of some common functional groups (2a – phenol, 2b-polyphenols, 2c-amine).

*Photochemical oxidation* is the basic case of photoionization, in which a molecule loses an electron to form a radical cation which is very reactive. Radical products can be formed in non-light catalysed reactions, but there is often a relationship between the non-photochemical oxidability of the compounds and their photochemical reactivity. Another class of photochemical oxidation is photooxygenation, in which light excitation catalyses radical addition. Phenols can be photochemically oxidized to quinoid oxidation products, which then form a peroxide adduct with air oxygen (Figure 2a). Polyphenols, e.g., pyrocatechinic derivatives are oxidized to quinones (Figure 2b) and aromatic primary amines to azo and nitro compounds (Figure 2c).^8^

## The role of bonds in photo reactivity

### Photodissociation processes

For photosensitizing drugs, the single bonds present in the molecule that have a low homolytic dissociation energy play an important role, since this energy is required for the bond to split equally into two radical moieties. In contrast, hydrolytic decomposition, where heterolytic bond splitting occurs, results in electron pair migration, anionic and cationic products, or intermediates. The energy of the two types of bond splitting is different.

Among the group of highly degradable chemical bonds we can mention C-Br, which has dissociation energy of 68 kcal/mole, C-I 53 kcal/mole, N-N 66 kcal/mole or O-O 51 kcal/mole. A drug compound which has in its structure one of these groups will be probably photosensitive, because these bonds does not require high energy to split.^19^

Double bonds play an important role in the photochemical reaction. Under the influence of light, the molecules are excited, their electron distribution, geometry changes, and transformations that are not typical in the normal state molecules occur.^32^

In the case of olefinic bonds or double bonds in the carbonyl group, one of the electrons on the π path is excited to the π* relaxation path during excitation, resulting in unpaired electrons. In the case of the carbonyl group, π-π* excitation may occur at the same time, which requires less energy than the π-π* excitation.

**Figure 3:**
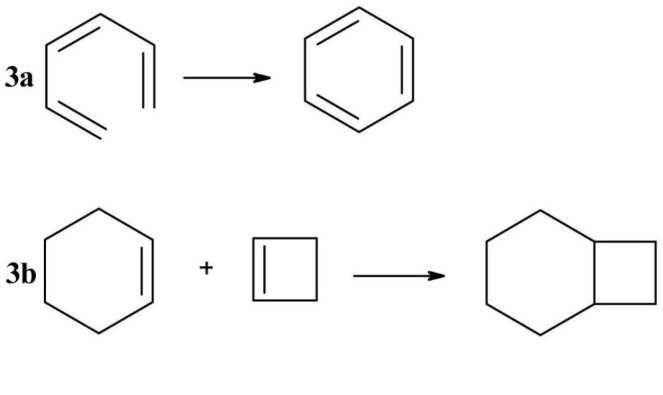
Examples of pericyclic reactions (3a - electrocyclization, 3 b - photocatalyzed cycloaddition).

Pericyclic reactions are characteristic to conjugated double bond molecules and are generally light catalysed. Such a pericyclic reaction is *electrocyclization* (Figure 3a), in which a π bond becomes a sigma bond followed by a ring closure.^19,30^

In the photocatalyzed cycloadditions (Figure 3b), two π bonds will be transformed in two sigma bonds, thus forming a new ring.^19,30^

## Photochemical degradation of drugs

We can find examples of drugs susceptible to photodegradation in almost all classes of pharmaceuticals used currently in therapy as mentioned in Table 1. Below we have selected a few relevant examples.

**Figure 4:**
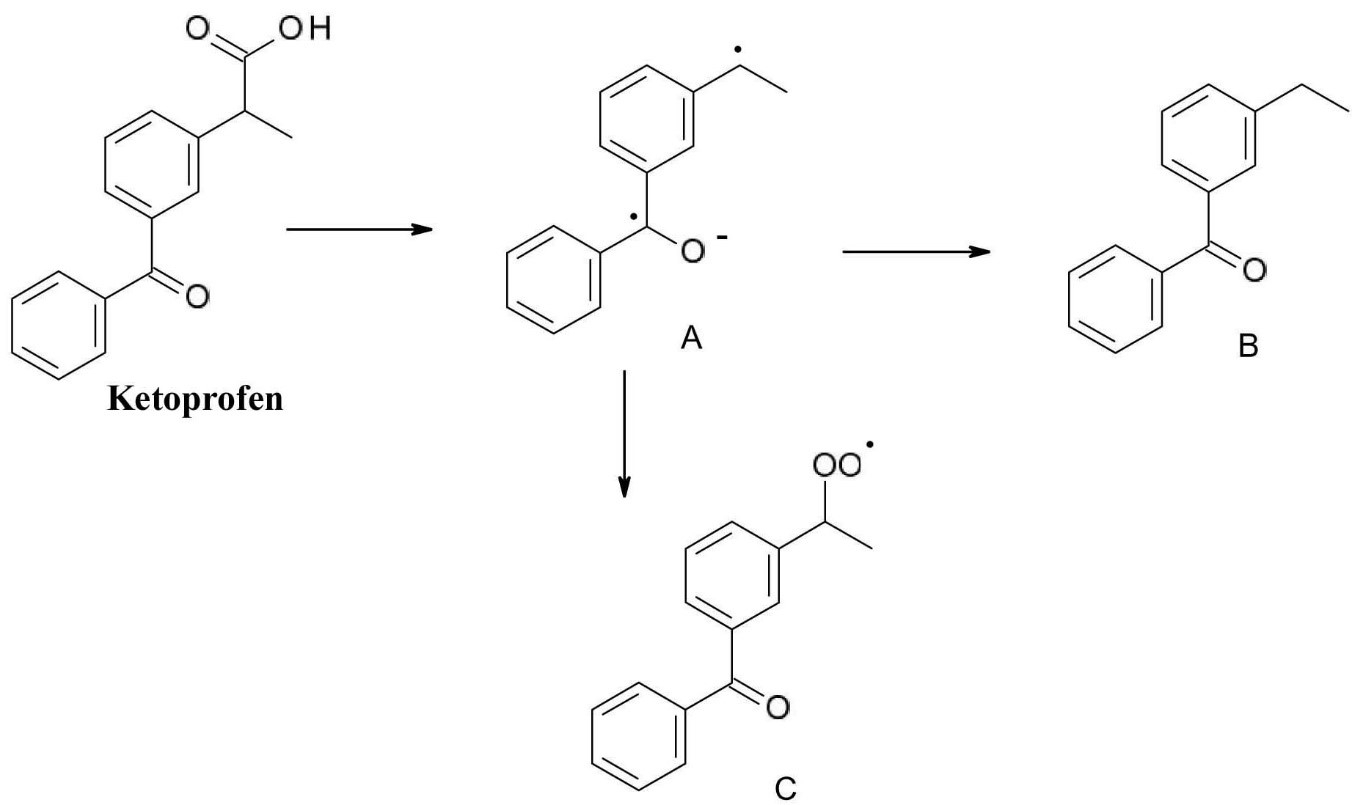
Photochemical degradation of ketoprofen.

*Ketoprofen*(2-(3-benzoylphenyl) propanoic acid)is one of the most frequently used aryl propionic acids type non-steroidal anti-inflammatory drug. Its characteristic decomposition product is compound (A) formed by photochemical decarboxylation which is further transformed by addition in compound (B) or by oxidation to peroxo radicals (compound C). It is worth to be mentioned that decomposition compounds (A) and (C) are toxic free radicals (Figure 4).^33^

*Benzodiazepin*e derivatives are used in therapy mainly as anxiolytics andare generally photolabile, but their degradation path depends on the structure of each derivative and on the degradation conditions.^34^

**Figure 5:**
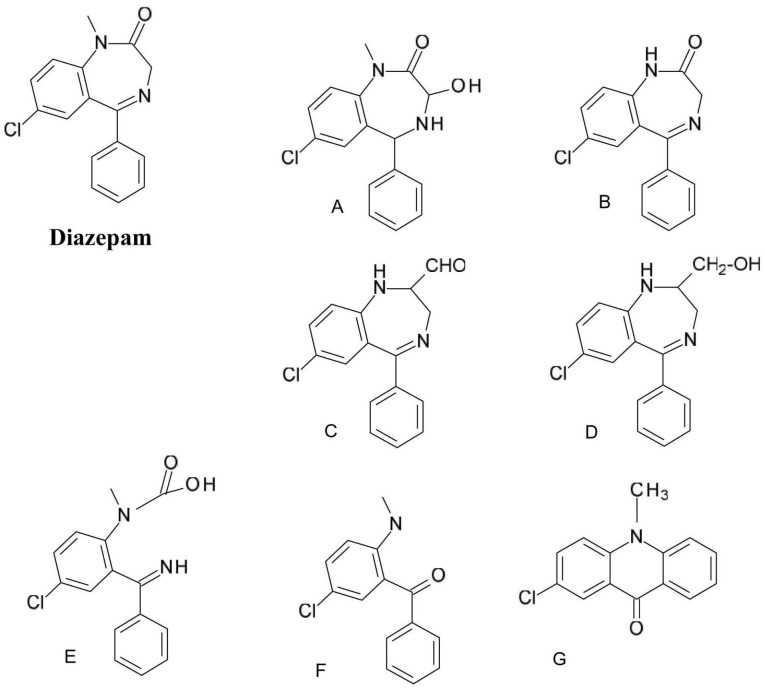
Diazepam photodegradation products.

A typical example of the photochemical reactivity of 1,4-benzodiazepines is the one of *diazepam* (7-chloro-1-methyl-5-phenyl-3H-1,4-benzodiazepin-2-one) decomposition (Figure 5). In the case of (A) and (B) degradation products the 1,4-diazepine ring system is retained; in product (A) a case photoaddition (water addition) and in case of product (B) N-CH_3_ bond photolysis occurs. In the case of products (C) and (D) the benzodiazepine skeleton photo isomerization through dihydroquinazoline (dihydrobenzopyrimidine) can be observed. Products (E) and (F) are formed by cleavage of a diazepine ring.^34^

**Figure 6:**
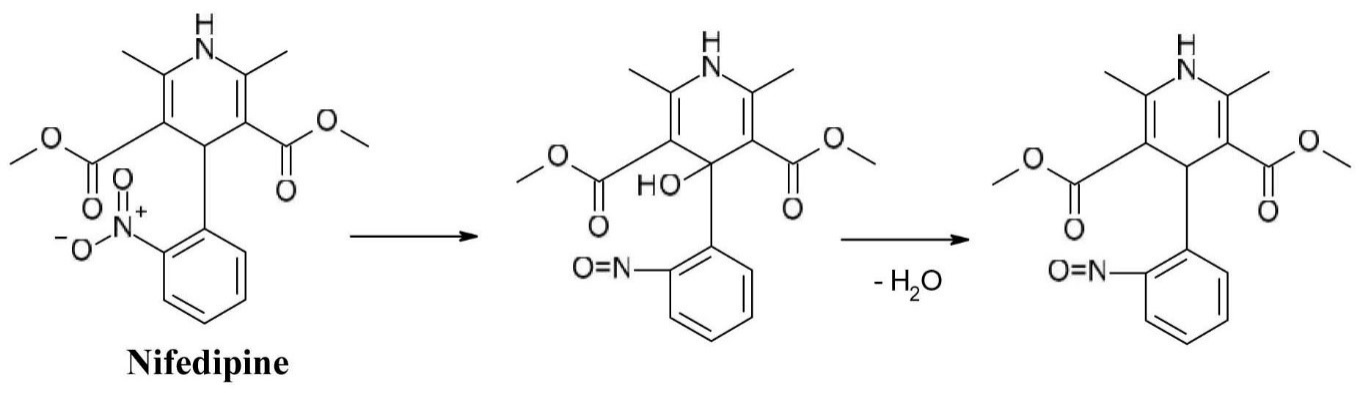
Photochemical degradation of nifedipine.

The photosensitizing properties of the nitro substituent of *1,4-dihydropyridine* derivatives with calcium channel blocker activity like *nifedipine* (dimethyl 2,6-dimethyl-4-(2-nitrophenyl)-1,4-dihydropyridine-3,5-dicarboxylate) are well known. Studies have shown that the position of the nitro group is strongly related the photo reactivity of the substance as 2-nitro substituted nifedipine has a significantly higher chemical reactivity than 3-nitro substituted nitrendipine. When exposed to light, the nitro group is converted to the nitroso group (Figure 6).^35^

**Figure 7:**
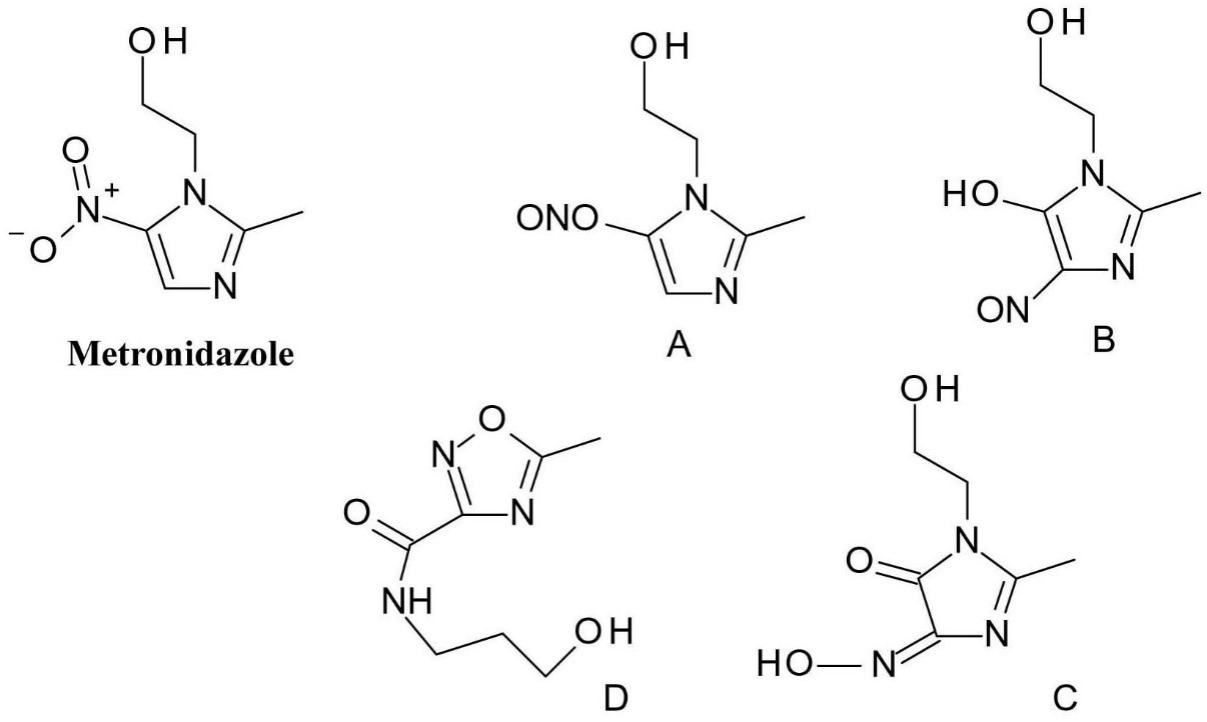
Metronidazole photodegradation products.

The photodegradation of *metronidazole* (2-(2-methyl-5-nitroimidazol-1-yl) ethanol) is based on the transformation of the nitro group in its structure which is reduced to a nitroso group (compounds A and B) as it was discussed earlier in the article (Figure 7). These intermediates then undergo further transformation and produce phenols. Further transformations can occur: as the phototautomerism of the derivative containing the nitroso group and the phenol group respectively can be observed (compound C), and an oxadiazole ring is formed, due to ring opening and subsequent rearrangement (compound D).^36^

**Figure 8:**
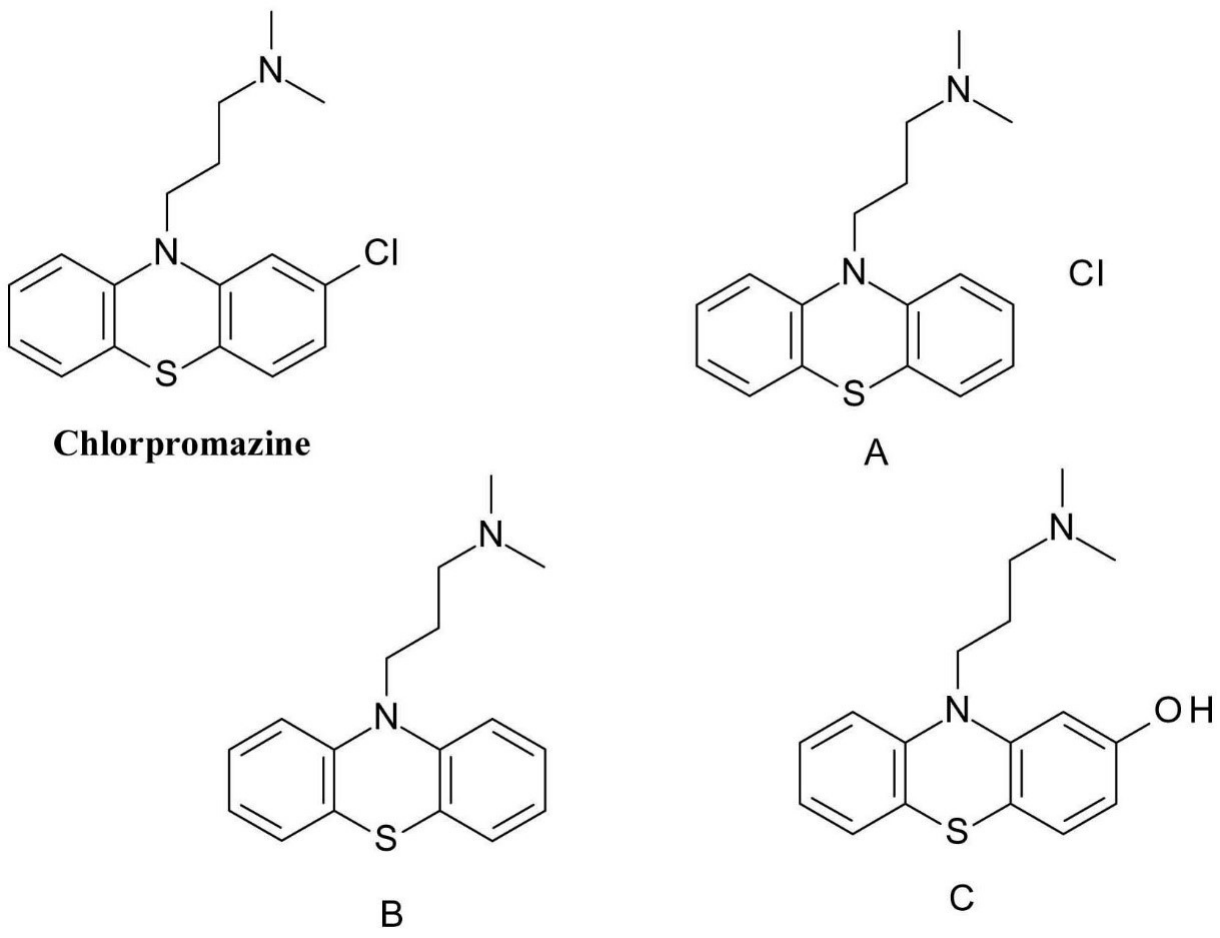
Chlorpromazine photodegradation products.

One of best-known drug phototoxicity examples is the one of the photodegradation of the phenothiazine neuroleptics like* chlorpromazine* (3-(2-chlorophenothiazin-10-yl)-N, N-dimethylpropan-1-amine) (Figure 8). The chlorine atom attached to the already highly oxidizable sulphur-containing phenothiazine ring is very easily removed by homolytic bond cleavage. The resulting phenothiazine radical respectively chlorine radical is highly reactive and can form a covalent bond with both proteins and bases of DNA nucleotides.^37^

Polycyclic fused ring systems containing heterocyclic rings and aromatic halogen (primarily chlorine) substituents are common among agents acting on the central nervous system (CNS), and in many cases photochemical reactivity occurs.^38^

**Figure 9:**
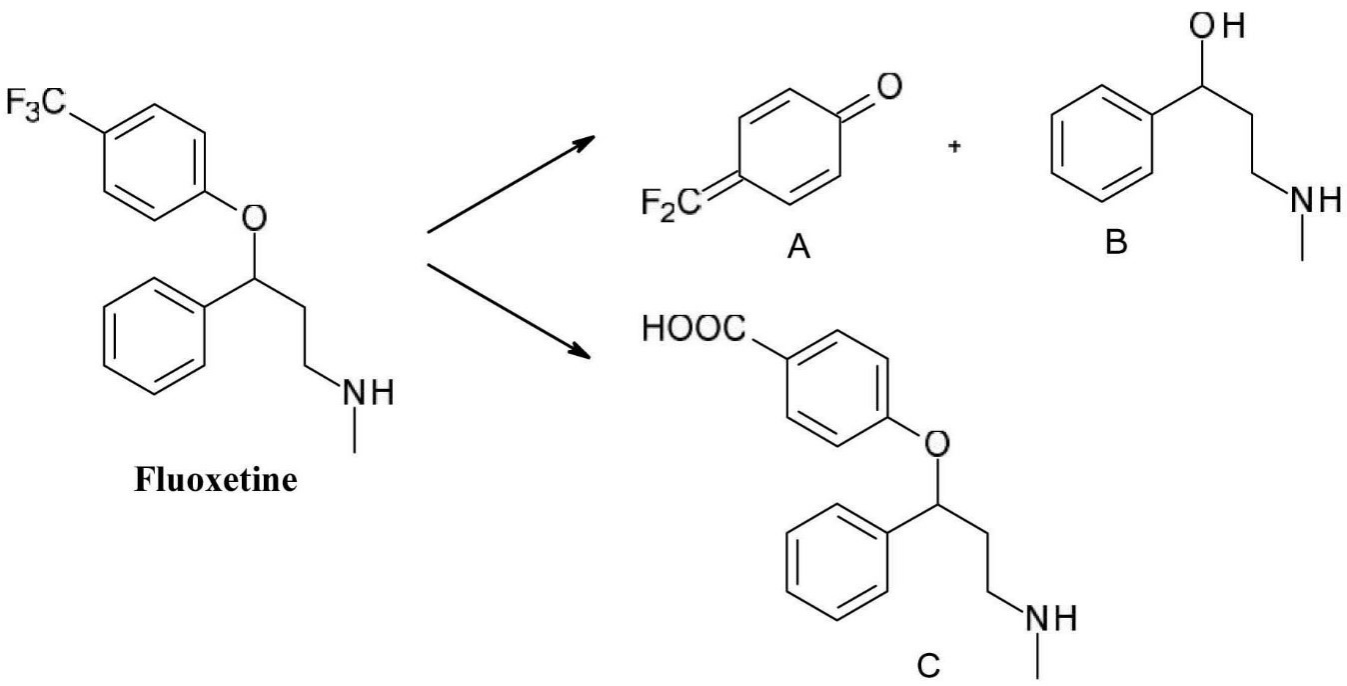
Photochemical degradation of fluoxetine.

In the case of the selective serotonin reuptake inhibitor antidepressant *fluoxetine*(N-methyl-3-phenyl-3-[4-(trifluoromethyl)phenoxy]propan-1-amine*)*, exposure to light can cause the degradation of the phenol ether bond and further oxidation of the para-trifluoromethyl phenol to form the quinoid product (compound A) and an alcohol (compound B). Another typical modification is the conversion of the trifluoromethyl group to a carboxyl group (compound C) (Figure 9).^39^

**Figure 10:**
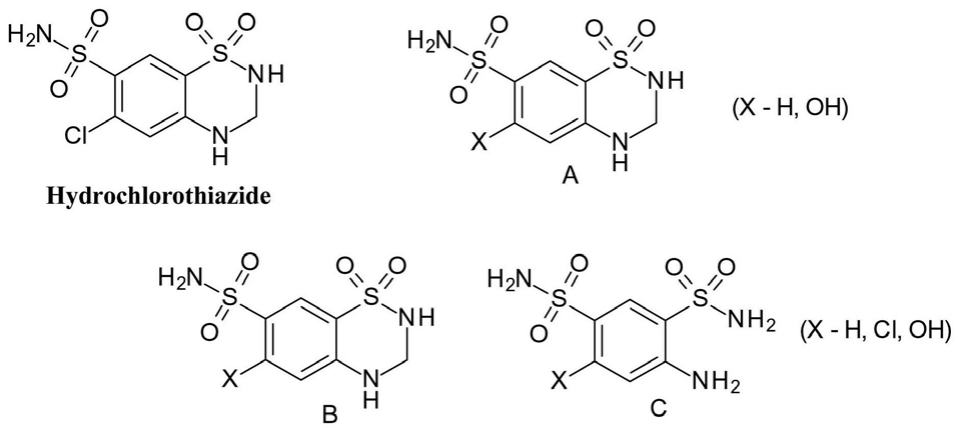
Hydrochlorothiazide photodegradation products.

The thiazide diuretic drug, *hydrochlorothiazide*(6-chloro-1,1-dioxo-3,4-dihydro-2H-1 λ^6^,2,4-benzothiadiazine-7-sulfonamide), is known to be photolabile under both aerobic and anaerobic conditions (Figure 10). The replacement of the chlorine substituent is one of the main processes (A), in addition, the dihydrothiazine ring may decompose (B) or oxidize to a thiadiazine ring (C).^40^

**Figure 11:**
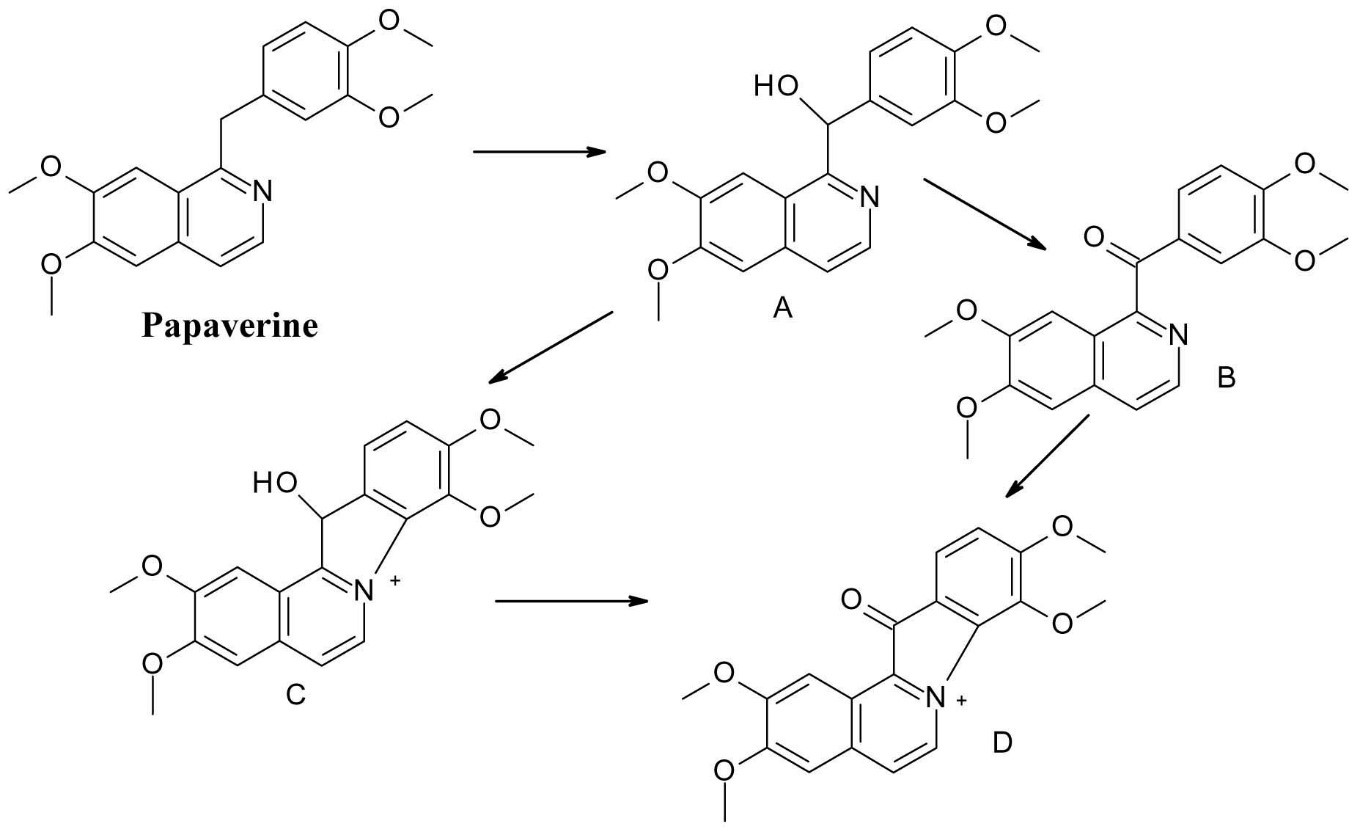
Photochemical degradation of papaverine.

The opium alkaloid spasmolytic drug *papaverine*(1-[(3,4-dimethoxyphenyl)methyl]-6,7-dimethoxyisoquinoline), is another molecule susceptible to oxidation, as the methylene bridge in papaverine molecule is vulnerable to oxidation (Figure 11). It is oxidized to a secondary alcohol – papaverinol (compound A) and subsequently to a ketone – papaveraldine (compound B), which is toxic. This decomposition is facilitated by light, and papaverinol respectively papaveraldine ring can be further transformed by condensation (compounds C and D).^41^

### In vivo reactivity of photochemical degradation products of drugs

The basic condition for light-induced drug toxicity is that the toxic molecules react chemically with the biomolecules through covalent bonds or convert them to a radical. Tissue damages are direct consequences involving specific organs (primarily skin), and one the other hand indirect effects also can occur, e.g., photoallergies.

The molecular mechanism of drug-induced photosensitization can take many different pathways, which can be divided into two different type of mechanisms: photosensitizing reactions via radicals (type I) and photosensitizing reactions via singlet oxygen (type II).^8,19^

#### Type I. mechanism

This mechanism can be further subdivided depending on whether the reaction is in the presence or absence of oxygen.

In this case of reactions in the presence of oxygen, four situations can be distinguished^8,19^:

- The excited molecule interacts with oxygen by energy transfer and form a complex. In polar solvents, this complex can dissociate to form a superoxide anion. This process is modest in efficiency since a direct interaction between the excited molecule and oxygen is more likely to lead to the formation of a single oxygen molecule.
- The excited molecule (M*) can interact with a biomolecule (M`) during electron transfer or uptake (depending on the redox potential of the M*/M` system) and form anionic radicals which, when reacting with oxygen, form a superoxide anion.
- Interaction between the substrate and the active drug molecule can also lead to radical formation (due to hydrogen depletion), which then reacts with oxygen to form peroxides.
- The excited molecule itself, can dissociate to form radicals that can convert the substrate into radicals or oxidize them by the oxygen in the air.

The first two reactions lead to the formation of superoxide anions, which are strong oxidizing agents and may be involved in further reactions.

The resulting superoxide anion, hydrogen peroxide and hydroxyl radicals have good tissue penetration due to their small size, and therefore are capable of extensive tissue damage.^8,19^

In an airy environment to avoid oxygen reactivity, four types of reaction pathways are distinguished:

- A redox reaction may occur between the toxic molecule and the biomolecule, which may subsequently produce free radicals.
- Hydrogen removal from the biomolecule accompanied by radical formation.
- Electron release in an aqueous environment.
- Compounds susceptible to photoaddition may react with nucleotide bases of DNA. As example we can mention the cytotoxicity of furanocoumarins, which is used therapeutically in dermatology in the treatment of psoriasis.

#### Type II. mechanism

Once formed, the singlet oxygen can further decompose by release of phosphorescence, collisions with solvent molecules, and photooxidation reaction with biomolecules, which include the formation of peroxides.

In the area exposed to light, the damage primarily affects the cell membrane components: lipids and proteins, which can lead to anaemia, lipid peroxidation, and photoreactive protein crosslinking; however, toxic molecules can also react with DNA nucleotide bases.^8,19^

## Study of photosensitization, clinical diagnostics

The Neutral Red Phototoxicity Method (3T3 NRU PT) is generally used to test phototoxicity as an *in vitro* test for soluble compounds.^42^

3T3 NRU phototoxicity test is designed to assess cytotoxicity of test substances in the presence or absence of UV light. Cytotoxicity is expressed as the concentration-dependent decrease in the uptake of the neutral red, measured 24 hours after treatment with test compound and irradiation. Neutral red is a weak cationic dye that readily penetrates the cell membrane and accumulates intracellularly in lysosomes. Chemical factors together with the irradiation may alter the cell surface and may result in a decreased uptake and binding of the neutral red dye. Differences in this uptake can be measured by spectrophotometry, which allows the distinction and quantification between viable, damaged, or dead cells.^42^

Guinea pigs are commonly used to test for photoallergy *in vivo*, while GLP-compliant testing for photocarcinogenicity employs SKH1 bald albino mouse model.^43^

The European Dermatology Forum (EURODERM) has developed guidelines for clinical trials. (https://www.euroderm.org/edf/index.php/edf-guidelines/category/3-guidelineson-photodermatoses). If, in a patient, the symptoms may indicate a photosensitivity reaction, its medication history must be clarified.^44^

It is important to find out when the symptoms started, when the patient might have switched to a new medicine, exactly what medicines the patient is taking, and so on. One method of detecting the photosensitizing effect of different drugs is photo-testing, in which the minimum erythema dose (MED) is measured using artificial UV-A and UV-B light while the patient is taking/not taking the medication.^3,45^

Another method used to diagnose photodermatitis is called photo patch testing.^3,43,45^ In doing so, the drug is applied topically on the patient’s skin, and the skin is covered for 24 hours. One half of the layer is then exposed to UV-A radiation, which at low doses below MED does not cause phototoxic reactions, while photoallergy does.^3^ After an additional 24 hours, the skin is checked, and if there is only a change on the side exposed to the light, it is a light-induced drug reaction. A similar method is photo scratch testing, in which the drug is not applied to the surface of the skin but is inserted into the deeper layers with a needle.^45^

## Conclusion

Drug photosensitivity is a common phenomenon and may involve a large variety of mechanisms, however the prevalence of photosensitivity in the general population is uncertain.

Drug-related photosensitivity refers to the development of cutaneous reactions due to the combination of chemical and light effects. Separate exposure to either the drug or to light alone is not sufficient to generate a reaction; however cutaneous manifestations can occur after photoactivation of the chemical.

Photosensitivity reactions may be classified as phototoxic or photoallergic; however often these two patterns overlap, making difficult to differentiate between them.

Data suggest that many drugs from different therapeutic classes are reported to be photosensitizers, however in many cases the relationship between the drug and light exposure is not clearly clarified. To take appropriate measures to prevent adverse effects, it is necessary to establish the relationship between chemical structure, chemical properties, and possible photodegradation of the drug.

Photosensitive drugs have chromophores that absorb photons in exogeneous form, being activated and undergoing chemical reactions upon sun exposure. The chemical structure of the chromophore will define the radiation of wavelength it will absorb.

Photosensitivity caused by medication should be treated as a diagnostic hypothesis any time a patient shows a skin eruption and a history of drug administration combined with light exposure. In addition, diagnostic tools, including photo-patch testing and photo-testing, may help in determining the causal agent.

## Ethical Issues

Not applicable.

## Conflict of Interest

The authors have no conflicts of interest to declare.
